# A Mixed-methods Study of How to Improve Primary-secondary Transitions in England: Combining Academic Research with Policy and Practice Consultation

**DOI:** 10.1007/s10648-026-10174-z

**Published:** 2026-07-04

**Authors:** C. L. Bagnall, D. Edge, E. Banwell, R. Seymour, N. Garnett, C. Mason, M. Panayiotou, D. Jindal-Snape, R. Packer, K. Hodgkin, C. Skerritt, C. Donaldson, J. Birchwood, A. McAreavey, E. Cunningham, J. E. Symonds, A. Holliman

**Affiliations:** 1 Manchester Institute of Education, University of Manchester, Oxford Road, Manchester, England M13 9PL UK; 2 Liverpool John Moores, Liverpool, England; 3 University of Dundee, Dundee, Scotland; 4 Cardiff Metropolitan University, Cardiff, Wales; 5 Trinity College Dublin, Dublin, Ireland; 6 Cardiff University, Cardiff, Wales; 7 University of Birmingham, Birmingham, England; 8 Manchester Metropolitan University, Manchester, England; 9 University of Waikato, Waikato, New Zealand; 10 University College London, London, England

**Keywords:** Primary-secondary school transitions, Educational policy, Co-production, Emotional wellbeing, Measurement, Intervention

## Abstract

Primary-secondary school transitions are critical periods in children’s educational and emotional development, which pose heightened risk for poor attainment, attendance, social adjustment, and mental health, particularly for vulnerable children. Yet, the multiple challenges presented during this time remain difficult for English schools to manage without clear statutory guidance. Government reports consistently identify transitions as a systemic weakness, while practitioners report uncertainty about what to prioritise, resulting in highly variable, locally interpreted provision. It is important that best practice guidance is developed, drawing on evidence from research, policy, and practice, to inform an evidence-informed transitions strategy in England. To narrow this gap, the present research takes an exploratory sequential mixed-methods design, triangulating multiple stakeholder perspectives and multidisciplinary evidence to develop an evidence-based framework with policy recommendations. A systematic literature review was first conducted to explore key priority areas identified within existing research. Building on these insights, 10 round-table discussions were conducted to aggregate multi-disciplinary perspectives of 52 experts within educational practice, research, and policy, nationwide. Data were analysed using Thematic Framework Analysis, to identify barriers and facilitators in implementing high-quality primary-secondary school transitions provision, and what key priority areas should be included in national strategy. Meta-inferences were then drawn to develop an evidence-based framework outlining five policy recommendations, with implementation proposals of how each might be enacted in practice. This research offers a conceptual foundation to inform policy and could have practical utility for informing high-quality research and practice. Practical and contextual challenges associated with introducing a statutory transitions strategy in England are considered.

## Introduction Statement

Primary-secondary school transitions mark a critical period in a child’s educational and emotional development (Symonds et al., 2023). In practice, the multiple challenges that primary-secondary school transitions present (e.g., academic discontinuities, relationship disjunctions, environmental, and identity changes), can be difficult to manage, especially without clear and consistent statutory guidance (Bagnall et al., 2021a). Government reports in England have consistently identified primary–secondary school transitions as poorly managed and a systemic weakness (DfE, 2026; Ofsted, 2015), and educational practitioners have raised not knowing what to prioritise, how, and when (Bagnall et al., 2024a). This has resulted in support for transitions being locally interpreted by schools and Local Authorities, leading to variation in quality and implementation, under-resourcing, and/or shared gaps, which remain inconsistently addressed, underdeveloped, and overlooked (White, 2020).

This inconsistent support for transitions is especially concerning recognising the body of evidence demonstrating the detrimental impact poor experiences of educational transitions can have on children’s academic attainment (West et al., 2010), attendance (Jindal-Snape et al., 2020), social adjustment (Harris & Nowland, 2021), and mental health (White, 2020), especially for more disadvantaged groups (Jindal-Snape et al., 2023). Thus, it is important that best practice guidance is developed and disseminated, drawing on evidence from research, policy, and practice, that could help guide an evidence-informed, statutory primary-secondary school transitions strategy in England.

To narrow this gap, and to provide an evidence-informed, statutory transitions strategy, the present research takes a rigorous exploratory sequential mixed-methods design. First, a systematic literature review (Study 1) was conducted to explore key priority areas identified within existing research. Building on these insights, 10 round-table discussions (Study 2) were conducted to aggregate multi-disciplinary perspectives of 52 experts within educational practice, research, and policy, nationwide. Findings from Studies 1 and 2 were then integrated, supporting complementarity and the generation of robust meta-inferences, synthesised as an evidence-based framework. This is accompanied with actionable policy recommendations to improve primary-secondary school transitions in England. Alongside offering a conceptual foundation that could inform policy, these recommendations aim to have practical utility in supporting the development, implementation, and evaluation of robust primary-secondary school transitions research, and high-quality practice, while seeking to address some of the limitations within the field, and contribute methodological insights.

## Background

### Significance of Primary-secondary School Transitions

Transitions from primary school to secondary school, which children typically negotiate at age 11 in England, marks a pivotal stage in a child’s educational and emotional development. During this time, children navigate multiple simultaneous changes, or as recognised in Jindal-Snape’s (2016, 2023) *Multiple and Multi-dimensional Transitions Theory (MMT)*, “transitions” in their school environment (including systemic structural, physical, and human aspects of change); social interactions (with both classmates and teachers); identity (primary/secondary school child, child/young person); and academic expectations. Primary-secondary school transitions are also nested amongst other developmental transitions experienced during early adolescence, such as the onset of puberty (Ng-Knight et al., 2016); marked changes in cognitive and emotional brain development (Blakemore & Mills, 2014); social and psychological changes (Moore et al., 2021); and school-based pressures, including academic national Standard Assessment Tests, and secondary school choice decisions in England (Bagnall, 2020). For this reason, researchers have advocated for primary-secondary school transitions to be examined through a multifaceted lens, to fully recognise the interconnected contribution of complex changes occurring for children, and significant others within their ecosystem (e.g. parents, teachers). While many children feel optimistic about the new opportunities they will have at secondary school, a substantive body of research also shows that this time is often accompanied by feelings of stress, anxiety, and declines in emotional wellbeing (Rice et al., 2021), which can begin approximately two years prior (Bagnall et al., 2024a) and can continue two years into secondary school (Jindal-Snape & Cantali, 2019). Poor experiences of primary-secondary school transitions are associated with low educational attainment, attendance and mental health at age 15 (Moore et al., 2020; West et al., 2010). These outcomes can exacerbate existing social inequalities, especially for more vulnerable children, such as those with special educational needs (Bagnall et al., 2021b), adverse childhood experiences (Donaldson et al., 2023); in receipt of Pupil Premium Funding (a government grant given to schools in England to support pupils from low socio- economic status backgrounds) (Garner & Bagnall, 2025), low academic attainment (Moore et al., 2021), and have been or are at risk of exclusion or suspension (Dunnett et al., 2025). Children with one or more of these characteristics are disproportionately impacted by poorly managed transitions (Donaldson et al., 2025). Thus, from a public health perspective, primary-secondary school transitions present a critical entrance point for early-intervention support to reduce (or prevent) associated health, social, and economic burdens.

### Policy Context for Primary-Secondary School Transitions in England

The multiple and disparate challenges that primary-secondary school transitions present can be difficult to manage, especially without clear and consistent policy frameworks with statutory requirements and guidance documents. Over the past decade, Government reports in England have consistently identified primary–secondary transitions as a period ‘not handled well’ (Ofsted, 2015, March, p. 65), with the quality of transitions practices between Key Stage 2 and Key Stage 3 ‘too often poorly managed’ (Ofsted, 2015, September, p. 21), ‘weak in over a quarter of the schools visited’ (Ofsted, 2014, p. 21), and it was concluded that ‘too many students are let down in the early stages of secondary school’ (Ofsted, 2015, p.1).

More recently, the Department for Education have reiterated that ‘a greater focus on transition periods in children and young people’s lives is needed’ (DfE, 2018, p.13), especially following COVID-19, which has shown the negative impact of primary-secondary school transitions on children’s emotional wellbeing to heighten (Bagnall et al., 2022). There is evidence to suggest that schools are still recovering (Garner & Bagnall, 2024) with primary–secondary transitions continuing to represent a systemic challenge (DfE, 2025), with many children believed to be ‘lost in transition’ (Children’s Commissioner, 2024, p. 96).

This is in stark contrast to other devolved nations in the United Kingdom (i.e., Scotland, Northern Ireland, and Wales) which have legislation in place to support primary-secondary school transitions. Although the devolved UK nations have established a range of transitions related policies and practices, these approaches are not readily transferable to the English context. In Wales, for example, the transitions process is underpinned by statutory duties mandating every secondary school and its cluster primaries to jointly produce an annual Transition Plan, as set out in the Transition from Primary to Secondary School (Wales) Regulations 2022 (Gov.uk, 2022). These plans are explicitly tied to Curriculum for Wales (Welsh Government, 2022) and must detail continuity of learning, cluster level curriculum planning, and support for learner progression and wellbeing. In Scotland, transitions support is embedded within a broader statutory framework for Additional Support Needs. Local Authorities must begin transitions planning twelve months in advance and work with education, health and social work services to coordinate continuity of support, as required by the national Supporting Children’s Learning Code of Practice. This creates a legally structured, multi-agency approach to primary-secondary school transitions, particularly robust for pupils with Additional Support Needs. In Northern Ireland, transitions are shaped by a centrally managed post-primary transfer system, with formal admissions timetables, clearly defined criteria, and structured parental guidance issued annually by the Education Authority; pupils with statements of special educational needs have a separate, fully coordinated transitions pathway managed directly by the Education Authority.

Collectively, these arrangements reflect systems in which transitions are embedded within national regulatory frameworks, supported by coordinated local structures, and more unified oversight. By contrast, England lacks comparable mechanisms of governance and institutional coherence. While Wales, Scotland, and Northern Ireland have increasingly prioritised collaborative approaches, emphasising inter-school cooperation, professional trust, and shared responsibility within lower-stakes accountability environments, England retains a top-down highly centralised structure with a heightened emphasis on measurable performance outcomes, including examination results and inspection judgements. England’s more fragmented governance landscape, disparities in resource allocation, and limited school-level autonomy, shapes what is feasible for school-transitions strategy development. Thus, while there is much to learn from devolved nations, England requires a strategy designed for its distinctive policy context and systemic conditions rather than a direct adoption of any existing model, which the present study will do to inform our recommendations.

### Limitations within Educational Practice

The lack of an evidence-informed, statutory transitions strategy in England has resulted in provision being locally interpreted by schools, Local Authorities, and other services, leading to variation in quality and implementation, under-resourcing, and/or shared gaps, which remain inconsistently addressed, and underdeveloped (White, 2020). Within educational practice, practitioners have raised not knowing what to prioritise over primary-secondary school transitions, how to most effectively intervene, and when, underscoring the need for evidence-informed statutory guidance (Bagnall et al., 2024b).

Taken together, a primary-secondary school transitions strategy, in England is urgently needed underpinned by (a) legislation and (b) evidence-informed recommendations. The former would ensure primary-secondary school transitions provision is, and remains, a priority, and more importantly a statutory responsibility that is embedded within broader educational standards, such as the Council for the Curriculum, Examinations & Assessment Key Stage 2 to Key Stage 3 transitions guidance in Northern Ireland (2022), which is embedded into wider school-improvement, wellbeing, and curriculum policy. Furthermore, legislation is critical in formalising accountability, enabling schools and Local Authorities to monitor transitions outcomes and refine practices accordingly; but also, in ensuring provision is well-resourced and embedded in school routines. Given that teaching staff are already overburdened by their workloads (Ozturk et al., 2025; Skerritt, 2021), time and resourcing as part of a planned, and supported process is imperative to ensure sustained commitment, and systemic-level change (Bagnall et al., 2024b; Donaldson et al., 2025), that is not reliant on individual staff or informal arrangements (Edge et al., 2024).

Ensuring primary-secondary school transitions provision is evidence-informed, and accessible to educators is vital. Whilst most academically developed and endorsed primary-secondary school transitions interventions efficacious, feasible to deliver, and suitable for universal and targeted populations within schools, have been synthesised and evaluated in academic literature; these evidence-based recommendations are not being accessed nor implemented by educators (Bagnall et al., 2024b). Instead, educators typically report using anecdotal sources to develop practices, as opposed to those based on research. This can lead to ‘patchwork practice’ across schools and Local Authorities leading to inconsistencies in the quality and scope of support offered (Growney, 2022). Thus, given that schools have limited time and monetary resources, educational practitioners need access to high-quality, evidence-informed primary-secondary school transitions approaches. However, the heterogenous focuses taken within primary-secondary school transitions research makes this difficult (Beatson et al., 2023) and it is important that researchers work collaboratively through co-production to ensure that practices are both evidence-informed and grounded in real-world practice (Bagnall & Jindal-Snape, 2023), which the present research will do.

### Limitations within Research

There has been extensive international systematic literature review research published in the past five years, which has synthesised primary-secondary school transitions research published since 2008. Together, these international systematic literature reviews have made significant contributions to theory and conceptualisation (Hannah et al., 2023; Jindal-Snape et al., 2021), instrumentation (Bagnall & Jindal-Snape, 2023), intervention design and evaluation (Beatson et al., 2023), and policy (Hodgkin et al., 2025). However, a notable gap within this research is the need to synthesise existing approaches used within educational practice to support children over primary-secondary school transitions and identify key priority areas, which we will address through the present research, through Study 1.

Furthermore, most existing systematic reviews on primary–secondary transitions rely heavily on qualitative evidence analysed using narrative synthesis, highlighting the need to also integrate quantitative and mixed-methods studies through meta-aggregation to provide a more holistic and methodologically robust evidence base. Additionally, existing systematic reviews on primary–secondary school transitions draw heavily on international evidence. While this has value, it poses limitations for informing education policy and practice in England, as educational systems and pedagogical philosophies differ widely across countries, which means findings from other contexts may not translate meaningfully. Thus, to ensure findings are relevant, actionable, and aligned with the unique features of the UK education system, a focused review on UK based evidence was conducted to provide policy makers, practitioners, and researchers with clear, transferable and ecological valid insights.

### Limitations within Education Policy

It could be argued that policy making is often based on the input of selected individuals and groups, often overlooking academic researchers and members of school communities (Skerritt, 2023a, 2026a). While within the academic research community school staff, students, and parents are not commonly considered policy makers (Skerritt, 2025, 2026b), some do consider policy to be ‘found in multiple sites, involving teachers, parents, and pupils as policy makers’ (Ozga, 2021, p. 298) and argue that ‘local policy change by invested individuals…can be meaningful and impactful’ (Hara & Good, 2023, p. 113). Thus, embedding co-production within educational research is essential to bridge gaps between research, policy, and practice, and to ensure that innovation is both evidence-informed and practice-led. Educational practitioners bring invaluable contextual expertise in understanding children’s needs, school cultures, and systemic barriers, which can help to refine conceptual, evidence-informed frameworks, ensuring that they are grounded in the realities of educational practice. This not only enhances the relevance, feasibility, and application of educational research and policy, but also empowers practitioners as active partners, fostering ownership, professional growth, and more effective implementation (Schildkamp, 2018). Therefore, extensive stakeholder consultation using both qualitative and quantitative approaches, has been conducted to identify the need, and inform the proposed research (Bagnall et al., 2020, 2021a, 2025b; Hodgkin et al., 2025; Jindal-Snape et al., 2023; Packer & Pierce, 2025; Packer et al., 2021). Building on this work, in Study 2, insights from a multi-disciplinary expert panel of 52 experts within educational practice, research, and policy, nationwide, were aggregated to understand existing barriers and facilitators in implementing high-quality transitions provision, and to establish key priority areas that should be included in a primary-secondary school transitions strategy in England.

### Current Study

In sum, despite widespread recognition of the importance of supporting primary-secondary school transitions, there is an absence of evidence-based, statutory guidance in England to improve this period. As mentioned earlier, this has led to fragmented support and variable outcomes for children, particularly those most vulnerable, who are disproportionately impacted by poorly managed transitions (Bagnall et al., 2025a; Donaldson et al., 2024). To narrow this gap, the present research developed an evidence-based framework with key recommendations to inform a primary-secondary school transitions strategy in England; a need which is timely, given the recent progression of the Children’s Wellbeing and Schools Bill in England (UK Parliament, 2025). As outlined above, there are valuable lessons that can be drawn from established primary-secondary school transitions policy, practice, and research across the other three UK nations. Our authorship team, which includes representation from all four nations, is well positioned to interpret these insights, in the context of informing policy and practice in England. Accordingly, evidence from across the UK will be synthesised to inform the development of an evidenced-informed primary-secondary school transitions strategy for England.

### Research Questions

In line with Brown (2014), we believe that the concept of evidence-informed policy making holds great promise; if policy makers make their decisions after considering multiple kinds of evidence, this increases the probability of a policy being more effective and more equitable for those most affected by it, as well as being more efficient in terms of its value for money. To do this, a deductive approach was first used, in Study 1, by conducting a systematic literature review to identify and synthesise evidence-based priority areas within existing UK research to improve primary-secondary school transitions, addressing Research Question 1:

1.1.1.1. What are the key priority areas identified within existing UK research to improve primary-secondary school transitions practice in the UK?
  Building on these insights, an inductive approach was then used, in Study 2, to aggregate professional views from 52 experts within educational practice, research, and policy, nationwide, through collection of primary data via round-table discussions, to address Research Questions 2 and 3:
2. What are the facilitators and barriers of “gold-standard” primary-secondary school transitions provision at the (a) child level; (b) family level; (c) school level; (d) Local Authority level; and (e) national level?
3. What are the key priority areas that should be included in a primary-secondary school transitions strategy in England, and what support, measurable objectives, and quality markers would be useful to implement this strategy?

It is argued that evidence-informed policy making is rarely achieved because researchers fail to present their findings in practical formats, that can be translated into real-world action (Hall & Brown, 2025). Of the view that ‘what is deemed good policy advice will be determined by how well it is communicated’ and ‘the best way to get traction is to communicate policy advice in clear and understandable ways’ (Skerritt, 2023b, pp. 758–759), this paper will present meta-inferences synthesised from the two strands of data collection, as a list of policy recommendations to be readily translated within a national primary-secondary school transitions strategy.

To do this an integrated, mixed methods sequential design, planned and made explicit from the start, was followed. Drawing on best practice within the field (Younas et al., 2025), and following Tashakkori and Teddlie (2010), credibility was established through design quality, with the systematic review informing and structuring the second study. The multi-informant approach, which gathered views from multiple stakeholders across several differing contexts, provided rigour and a truly holistic foundation for developing meta-inferences. In this way, policy recommendations are grounded in evidence that was integrated from numerous strands, rather than either strand alone.

Following Tashakkori and Teddlie (2008) and Schoonenboom (2022) the interpretation of meta-inferences was a two-stage process. In the first stage, findings from each study were analysed separately against the main research question. In the second stage, the findings from both studies were compared, contrasted and integrated. It was here that there was movement from strand-level to more global explanations of what “gold-standard” transitions provision entails. This stage involved the generation of relational meta-inferences, aligning with Younas et al.’s (2025) best practice guidance, whereby inferences drawn from the systematic literature review and the multi-informant stakeholder consultation were examined in relation to one another. By identifying conceptual linkages, points of convergence and divergence, and contingent relationships across these datasets, higher-order interpretations were developed that extended beyond the insights of either strand alone. It was through this relational analytic process that policy recommendations emerged. These recommendations were not grounded in either study but in the relationship between the systematic literature review and the multi-informant stakeholders’ perspectives.

## Study 1: Systematic Literature Review

A notable gap within this research is the need to synthesise existing approaches used within educational practice to support children over primary-secondary school transitions and identify key priority areas. To address this gap, a systematic literature review was conducted, to identify key priority areas identified within existing research. The heterogenous focuses taken within primary-secondary school transitions research makes this difficult, as do the differing methodologies, presenting the need to integrate qualitative, quantitative, and mixed-methods studies through meta-aggregation to provide a clearer understanding of what constitutes effective practice, where gaps persist, and which areas require policy attention. In doing so, the systematic literature review provides a critical and necessary foundation for developing coherent, evidence-informed guidance. As outlined above, the review synthesised research evidence from across the UK. This is recognising the valuable lessons that can be drawn from established primary-secondary school transitions practice in Scotland, Northern Ireland and Wales. Drawing on this broader evidence will support the development of insights that are more transferable, ecologically valid and capable of informing education policy and practice in England, while providing policy makers, practitioners and researchers with clear guidance.

## Method

The systematic literature review is based on the Evidence for Policy and Practice Information and Co-ordinating Centre’s (EPPI-Centre, 2010) approach for undertaking systematic literature reviews and adhered to Gough et al.’s (2012) seven step methodology. To maximise available data, this review combined qualitative, quantitative, and mixed-methods primary research. To enable this methodology, following quality and relevance assessment (outlined below), data transformation was required which included coding findings from the results, discussion, and conclusion sections of all included studies, using thematic synthesis, to develop descriptive themes. This is a reliable method that retains a transparent and clear link between the conclusions and original papers, while at the same time adhering to the principles of systematic reviews (Thomas & Harden, 2008). The systematic identification and synthesis of studies focused on Research Question 1 (RQ1).

### Search Strategy

**Fig. 1:**
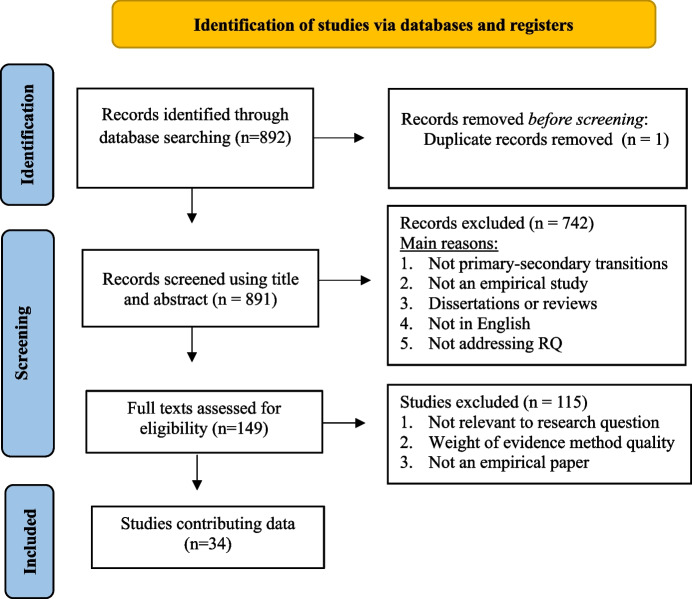
PRISMA flow diagram of study selection

Prior to conducting the review, the search strategy protocol was pre-registered with the International Prospective Register of Systematic Reviews (PROSPERO; registration CRD42023393117), outlining the research objectives, transparent pre-identified inclusion and exclusion criteria, comprehensive search strategy, and the analysis strategy, to enhance reliability and prevent selective reporting. The PRISMA flow diagram (see Fig. 1) outlines the process of searching and identifying relevant studies. The databases most relevant to the purpose of this systematic literature review included: Education Resources Information Centre (ERIC); British Education Index (BEI); Web of Science (WoS – Science Citation Index Expanded; Social Sciences Citation Index; Arts & Humanities Citation Index), PsycINFO, and Applied Social Sciences Index and Abstracts (ASSIA), which mirrors existing systematic literature reviews conducted within the field to-date, ensuring the systematic literature review builds on a strong foundation, and avoids omission of key evidence, bias, and misalignment with existing knowledge (Bagnall & Jindal-Snape, 2023; Jindal-Snape et al., 2020, 2021).

### Screening Studies

To reduce bias an extended research team screened the literature using the inclusion criteria (see Table 1). Cross-checking was employed at different stages to enhance the rigour of the process.

**Table 1:** Table of search terms and inclusion/exclusion criteria

Aspect	Criteria
Relevance	Relates directly to the research question as can be seen from the title, abstract or key words used in the paper
Search terms	1. Transition*, 2. Transition, 3. Mov* in combination with i. primary school, ii. middle school in combination with high school or secondary school
Period	Between 2008 and 1 October 2025
Participants	Children in mainstream education
Age-range	10–14
Geographical spread	UK only, with the country and educational context clearly stated
Research base	Empirical research, i.e. research that is based on observation and measurement of phenomena, as directly experienced by the researcher, be it qualitative, quantitative or mixed methods
Transparency	Methodology of the research should be explicit (e.g., sample size, instruments, analysis)
Reliability/validity	As far as can be determined, the findings upon which the review is based must be valid and reliable, taking into account the type of study, the degree of synthesis and interpretation versus descriptive for qualitative research, mitigating bias. The study must have been peer-reviewed due to its perceived robustness

### Quality and Relevance Assessment

Transparency, consistency, and congruence between philosophical perspectives, theoretical frameworks, and methodology is integral to determine the robustness of a study and the interpretations drawn. This is vital to advance primary–secondary school transitions theory, research, policy, and practice (Hannah et al., 2023), and studies chosen for inclusion were screened for quality and risk of bias using the weight of evidence screening method developed by EPPI-Centre (2010). For inclusion, each study needed to have a minimum of a satisfactory score in each of the three criteria of the scoring matrix (see Table 2). Cross-checking was employed to enhance rigour.

**Table 2:** Table of weight of evidence scoring matrices

	Level/Criterion	Methodological quality	Methodological relevance	Topic relevance
1	Excellent	Excellent research design with clear justification of all decisions: e.g. sample, instruments, analysis. Clear evidence of measures taken to maximise internal and external validity and reliability and reduce sources of bias	Research questions (RQ) clearly stated. Methodology is highly relevant to their RQs and answers them in detail	Study is very closely aligned to the key review objectives and provides very strong evidence upon which to base future policy/action
2	Good	Research design clearly stated with evidence of sensible decisions taken to provide valid and reliable findings	RQs are explicit or can be deduced from text. Findings address RQs	Study is broadly in line with one of the key review objectives and provides useful evidence
3	Satisfactory	Research design may be implicit but appears sensible and likely to yield useful data	RQs implicit but appear to be broadly matched by research design and findings	At least part of the study findings is relevant to the key review objectives
4	Inadequate	Research design not stated or contains flaws	RQs not stated or not matched by design	Study does not address any key research objective

### Synthesis and Reporting

Data from the findings, discussion, and conclusion sections of the 34 included papers, were analysed using meta-aggregative synthesis, consistent with EPPI-Centre (2010) guidance. Extracted findings from included studies were first reviewed for similarity in meaning, and then organised into categories representing commonalities, which required an iterative interpretation of the data. These categories were subsequently aggregated into synthesised findings that addressed RQ1. Throughout the process, the analysis sought to preserve the intent and context of the primary data rather than reinterpret it, ensuring a transparent and auditable link between the original evidence and the final synthesis. A meta-aggregative approach was selected, as the review sought to identify and summarise practical strategies, aligning with an aggregative orientation that integrates evidence to produce actionable, practice-focused conclusions and recommendations. Extracted data was analysed using inductive thematic synthesis, taking an iterative approach, to develop descriptive themes from which analytical themes, identifying key priority areas were subsequently developed (Stern et al., 2020). All data were initially coded, with the codes grouped together to form descriptive themes. The descriptive themes were closely related to the underlying codes and data (see Table 3).

**Table 3:** Table of descriptive themes and resultant analytical themes weight of evidence scoring

Descriptive themes	Theme title	Analytical themes
Making children familiar with their new school; school visits, joint projects, discussions, presentations, information etc., reduce unknowns and hence anxiety, emphasise positives; new friends, subjects and choices, conceptualise change	**Familiarity**	Making children familiar with their school to be; physical environment, staff, children, thus reducing the unknowns and hence anxiety, ensuring children have a balanced expectation of the challenges to come and the opportunities for new friends, subjects and choices
Children need positive relationships with peers, staff and other adults for support, harder to develop pupil-staff relationship in secondary school	**Relationships**	Supporting children to create and maintain positive, supportive relationships with; peers, staff, families and other adults
Protective intrapersonal factors such as: problem solving, emotional control, ability to build relationships, adaptability, change results in revert to the familiar, need to be positive	**Intrapersonal skills**	Helping children to develop their skills in; problem solving, gaining emotional control, building relationships, adaptability and positivity
Pastoral support provided to the children in school, including emotionally available adults, safe places, peer mentors, family support, peer support, application of reasonable consistent rules, school ethos friendly, no one size fits all, ongoing continuous support, standardised wellbeing scale	**Pastoral**	Continuous support delivered by schools to ensure children feel; safe, supported, and respected (especially in secondary school), underpinned by both standardised wellbeing measures, and attention to the social environment and support during transitions, whilst reflecting the uniqueness of each child
Primary and secondary schools working together; need common understanding, need trust for data, need to be congruent with society, primary teachers' predictions are right, need collaboration and continuity, transition leaders create links	**Collaboration**	Primary, secondary schools and other bodies working together with; good communications, common objectives, common understandings, trust and in a respectful and courteous manner to the benefit of children; transition leaders create links
Families and carers; important for support, need to understand the processes, need involvement and communications with school	**Families**	Inclusion of families, wider family members and carers who need to; be well informed, be able to participate in the transitions activities, have good communications with schools, feel involved and able to provide meaningful support to children

## Results

Data from the 34 research papers (six mixed-methods, 13 quantitative and 15 qualitative papers) are presented below, separated under six themes, identified as key priority areas, to address RQ1. It is relevant to note that the six themes are not mutually exclusive in that there is overlap between themes, which reflects the fact that some codes contribute to more than one theme and that transitions are complex and multi-dimensional (Jindal-Snape, 2016).

### Familiarity

Familiarisation with the new school in a physical, academic, and social context is discussed as important in reducing anxiety. As primary-secondary school transitions are a systemic change, systemic solutions such as bridging projects appeared effective by providing exposure to new teachers and the environment in Year 6 (final year of primary school in England) and providing continuity into Year 7 (first year of secondary school in England) to minimise multiple changes being navigated at the same time (Neal et al., 2016; Rice et al., 2021).

#### Preparation in Primary School

Preparation at primary school is important in addressing fears and concerns prior to primary-secondary school transitions (Jindal-Snape & Cantali, 2019) with children who report greater concerns in primary school showing poorer adjustment in secondary school (Nowland & Qualter, 2020). References were made to the importance of discussing school transitions throughout the last two years of primary school to manage expectations, especially with parents/carers as well as teachers (Bagnall et al., 2020; Curson et al., 2019; Packer et al., 2021). Many transitions interventions were discussed (Bagnall et al., 2021a; Beatson et al., 2021), and role play was shown to be useful in encouraging children to explore their feelings, identify challenges and solutions, build self-esteem and empathy with other children, and in so doing reduce concerns (Barlow, 2021).

#### Secondary School Familiarisation

Secondary schools providing accurate, realistic, and balanced information was discussed as important in managing expectations, and reducing anxiety, as over promising can lead to dissatisfaction (Ashton, 2008), and the tendency to focus on the potential negatives can create fear and anxiety (Hammond, 2016). Ongoing familiarisation with the secondary school was highlighted as important for alleviating transitions-related anxiety (Curson et al., 2019).

## Relationships

### Peers

Most studies found that having good peer relationships were important in terms of having successful transitions experiences and is reported as being the children’s and parents’ main focus over and above their academic work (Bagnall et al., 2020). Peer relationships can foster self-esteem, wellbeing, and provide access to support and social networks; and many studies noted that transitioning with primary school peers helped children settle into secondary school (Evangelou et al., 2008). Equally several studies found that transitioning without peers could lead to difficult transitions (Brewin & Statham, 2011).

While in some studies primary-secondary school transitions was shown to lead to more and better quality peer relationships being developed in secondary schools due to a bigger pool of friends to choose from (Jindal-Snape et al., 2020), in other studies children were concerned about the potential loss of existing friendships and the difficulties in creating new ones (Nowland & Qualter, 2020). Studies reported that children missed their old relationships with peers and staff (Bagnall et al., 2020). The loss of existing friendships was reported as potentially leading to fear of abandonment/rejection, with children being scared and lonely which could lead to anxiety and depression (Hammond, 2016).

Studies also reported that school policies impacted upon the stability of children’s friendships and given the impact which friendship stability potentially has on child wellbeing and academic performance, suggests that schools would be well advised to focus on supporting friendships and promoting interpersonal skills (Ng‐Knight et al., 2019). The value of friendships and connecting with peer groups is such that schools could develop practices to promote their formation with drama and a broad range of extra curricula activities identified as effective in this regard (Barlow, 2021).

### Teaching Staff

Most studies reported the importance of positive pupil-staff relationships (Ashton, 2008; Brewin & Statham, 2011). A good relationship enabled the staff to be more supportive, creating a sense of belonging, promoting academic achievement and wellbeing outcomes (Mahmud, 2020). Studies also noted that it was harder for secondary staff to get to know children, and that staff were not seen to be as approachable as primary staff (Holt et al., 2023). There was also perceived to be a risk that staff focused excessively upon rules which could generate resistance from children (Brewin & Statham, 2011).

### Other Adults

The need for children to feel supported was emphasised by a number of studies (Bailey & Baines, 2012). In addition to children’s peers and school staff, support could be provided by families or indeed other trusted adults within the child’s community that are available, and willing to communicate (Holt et al., 2023). Ongoing, trusted relationships, such as those with family members, were seen as providing valuable continuity in a child’s rapidly changing life (Brewin & Statham, 2011).

### School Support Social Networks

Many studies also placed a responsibility upon secondary schools to develop and implement policies that impacted positively on children’s relationships. Findings suggested that interventions should focus on managing anxiety in social situations, which could assist in the formation of friendships (Nowland & Qualter, 2020). Studies noted that the formation of positive relationships with peers and staff within the school generated a sense of belonging which was seen as important in improving wellbeing (Francis et al., 2021).

## Intrapersonal Skills

Studies highlighted that children’s intrapersonal characteristics such as: problem solving, emotional control, adaptability, and optimism were important during transitions (Curson et al., 2019). The ability to self-regulate was identified in several studies as linking to successful transitions, with children experiencing less anxiety and better able to manage threatening situations (Bailey & Baines, 2012; Nowland & Qualter, 2020). Similarly, children’s adaptability, was considered to help them adapt to primary-secondary school transitions challenges, as was approaching transitions with optimism (Bailey & Baines, 2012). Conversely, those children who, pre-transfer, envisaged experiencing problems during transitions tended to have them, demonstrating the importance of building positive expectations, and evidence of self-fulfilling prophecy (Jindal-Snape & Cantali, 2019).

Several studies noted that while schools typically addressed emotional and social development through Personal Social, Health and Economic (PSHE) lessons, greater emphasis on these skills was needed given their importance (Holt et al., 2023; Mahmud, 2020). Suggestions included improving adaptability through school and parent/carer involvement, explaining expected changes and appropriate coping strategies (Holliman et al., 2020). Additional education on emotional regulation was also proposed to reduce social anxiety and support the formation of new friendships (Nowland & Qualter, 2020).

## Pastoral

### Ongoing, Continuous Support

Studies emphasised the importance of pastoral support provided to children by schools throughout Year 6 and Year 7. It was proposed that transitions concerns should be addressed throughout the last year of primary school and continue through the first year of secondary school (Bagnall et al., 2024a; Curson et al., 2019).

### Social Environment and Support

The secondary school social environment was identified as critical, with a need for supportive and approachable staff and for school rules to be applied consistently yet flexibly as children settled in (Bagnall et al., 2020). Support from empathetic staff and older pupils was associated with increased confidence, self-esteem, and social connectedness, offering protection against transitions-related risks (Evangelou et al., 2008). Although there was consensus that support must be flexible and responsive rather than “one size fits all,”. Provision was often reactive in secondary school, relying on pupils to seek help (Bloyce & Frederickson, 2012). Support practices varied across schools and included taster days, peer mentoring, and extracurricular activities, which promoted inclusion and belonging, while support staff played a key pastoral role due to their greater availability and approachability.

### Standardised Wellbeing Scale

Primary-secondary school transitions can be a challenging time for all children, many of whom have an overall positive experience, but a significant minority can have a negative experience which can have negative short and long-term implications (Beatson et al., 2023). There are a range of methodologies and strategies which schools use to support pupils over this time, however there is a need for a standardised and validated instrument to assess children’s wellbeing which enables educators to monitor the efficacy of these interventions (Bagnall et al., 2024a; Beatson et al., 2023). As primary-secondary school transitions are triggered by multiple, interconnected changes, over an extended period, any instrument must be standardised, sensitive, reliable, and suitable for longitudinal use (Bagnall et al., 2024b). In the absence of such an instrument, it remains challenging to robustly evaluate interventions and identify those that are most effective.

## Collaboration

### Between Schools

Studies concluded that increased collaboration between primary and secondary schools would be beneficial for all stakeholders (Bagnall et al., 2020). Although schools share pupil data, secondary schools often question its accuracy (Evangelou et al., 2008), partly due to differing views on its purpose: primary schools use it to demonstrate outcomes, while secondary schools view it as a baseline for measuring progress. Developing joint resources on pedagogy, assessment, and curriculum could strengthen continuity between schools (Holland et al., 2019).

### Between Stakeholders

Additionally, there are differing views between stakeholders (e.g., children, parents, school staff and other professionals drawn from governmental and academic bodies) regarding whether transitions are: a one-off event or a process, or series of processes over time (Holt et al., 2023). A shared conceptualisation of transitions is fundamental in developing best-practice (Jindal-Snape & Cantali, 2019).

### Transition leaders

Studies noted that Transition Leaders have a central role in co-ordinating collaboration between schools and stakeholders over primary-secondary school transitions. By overseeing the transitions process, they offer continuity for children, as they will likely have visited the primary schools and possibly met with individual pupils. They facilitate trust and understanding between primary and secondary phases through direct engagement with schools and individual children. However, their effectiveness depends on having sufficient flexibility and resources, which are not always available (Bagnall et al., 2024a).

## Families

A key source of support for children during transitions was their family, including immediate and extended family members (Hodgkin et al., 2025). Children turned to their family members and friends to discuss potential or actual problems, and to obtain support more than they turned to school staff (Bloyce & Frederickson, 2012). While open family discussions about transitions are important, these conversations should be child led and balanced, avoiding undue emphasis on potential concerns such as loss of friendships and instead highlighting benefits to prevent unnecessary anxiety (Bagnall et al., 2020).

Families could provide support by being involved with, and aware of the processes, explaining differences between schools and by suggesting coping mechanisms which children may use, and in so doing reduce anxiety (Curson et al., 2019). However, to enable families to respond with effective support, it is essential that they are informed and included within the planning and preparation of transitions, recognising they have knowledge of their child beyond the context of the school. For this to occur good open communications between the schools, children and families are critical, which can be dependent on families own experiences of school transitions.

## Study 2: Roundtable Discussions

Embedding co-production within educational research is essential for bridging the persistent gaps between research, policy, and practice. This is integral in providing invaluable contextual knowledge, which ensures that evidence-informed frameworks are grounded in real-world practice, feasible to implement and more likely to achieve sustained impact. Co-production also strengthens practitioner ownership and supports more effective implementation (Schildkamp, 2019). Recognising this, the present research collected insights from a multidisciplinary panel of 52 experts across educational practice, research and policy.

Building directly on the systematic literature review themes, identified in Study 1, particularly the necessity of taking a system-level perspective, Research Question (RQ) 2 takes a more detailed insight into the dynamics of primary-secondary school transitions across the interconnected ecological levels. By explicitly examining how constraints and enabling conditions operate within and across these levels, RQ2 is designed to generate robust, actionable insights with direct relevance for policy, practice, and future research. These system-level insights are then synthesised by RQ3, to identify substantive priority areas requiring strategic attention at national level, grounded in multi-level evidence. Collectively, these RQs are designed to generate a robust, evidence-informed foundation to inform the development, implementation, and evaluation of a national primary-secondary school transitions strategy.

As outlined above, there are valuable lessons that can be drawn from established primary-secondary school transitions policy, practice, and research across the other three UK nations. Accordingly, experts from across the UK participated in the 10 roundtable discussions, to synthesise views within primary-secondary school transitions educational practice, research, and policy, nationwide, to inform the development of an evidenced-informed primary-secondary school transitions strategy for England.

## Method

### Delegates

On Friday 24th January 2025, a Primary-Secondary School Transitions Strategy Event was held, to solicit and aggregate expert views from a multi-disciplinary expert panel of 52 experts within educational practice (Local Authority leads *n* = 13; school teachers *n* = 11), research (academics *n* = 11; professional practice *n* = 8), and policy (*n* = 9), nationwide (England *n* = 45, Scotland *n* = 3, Wales *n* = 2, Northern Ireland *n* = 2), through 10 round-table discussions. The delegates were recruited from our research centre’s transitions network, existing school networks across the UK (including Initial Teacher Education programmes), along with snowball sampling via our primary-secondary transitions advisory group to ensure that we had a broad and representative sample; this is in line with recommendations from similar research in the field (Bagnall et al., 2025a; Symonds et al., 2022). Data collected in the round table discussions were anonymised.

### Materials

Roundtable discussion guides were prepared in advance, which were informed by extensive prior stakeholder consultation (Bagnall et al., 2020, 2025a; Hodgkin et al., 2025; Jindal-Snape et al., 2020; Packer & Pierce, 2025). A semi-structured design was followed to allow the discussions to be guided by the delegates, and for us to probe further into the issues raised, pertinent to answer our research questions.

### Procedure

Each delegate contributed to two round-table discussions, with eight or nine other delegates and one research facilitator. The first-round table involved delegates discussing, at their tables, the facilitators and barriers of “gold-standard” primary-secondary school transitions provision, at five levels (pupil, family, school, Local Authority, and national) that progressed in size from the individual to the proximal systems that support them, to wider distal systems that control the processes within these smaller systems. The term “gold-standard” was the wording used in the study materials presented to delegates and is retained, enclosed in quotation marks, in this section and the analysis section to accurately reflect that framing. To ensure consistency and shared understanding across tables, delegates were provided with an explicit definition of "gold-standard" primary-secondary school transitions provision prior to discussion, which was defined as high-quality support that is evidence-informed, rigorous, and comprehensive.

The second round-table then focussed on key priority areas that should be included in a primary-secondary school transitions strategy in England, and what support, measurable objectives, and quality markers would be useful to implement this strategy. As a group delegates made shared written-notes summarising their discussions.

### Ethics

Delegates were expert contributors within their professional capacity, and the study involved consultation on professional practice and co-design processes. The University of Manchester’s Research Ethics Committee (UREC) confirmed that formal ethical approval was not required for this stakeholder consultation activity. For good practice, we ensured all procedures adhered to the British Psychological Society’s *Code of Human Research Ethics*, ensuring transparency, informed consent (including providing consent to be audio-recorded and for publication of anonymised insights through a signed consent form), and secure data management. The discussions were audio recorded, and sent to a university-approved transcription service, and then this was analysed by the core research team, who were all attendees at the event. Delegates have not been named within the current, or any other, publications.

### Data Analysis

***Written data*** collected during the roundtable discussions, were analysed first using inductive content analysis by the first and last author, following Elo and Kyngäs’s (2008) three phase process: preparation, organising, and reporting. During the preparation phase, both authors immersed themselves in the textual data from all roundtable discussions through repeated reading, to become familiar with the content, and begin identifying preliminary insights. In the organisation phase, the authors conduced line-by-line open inter-coding to extract significant features of the content, which were cross-checked to establish credibility and confirmability. Similar codes were then grouped into subcategories and following abstraction clustered into higher-order conceptual main categories that described broader patterns and meanings in the data. This phase was iterative and involved moving back and forth between the data and emerging categories to refine meanings and ensure consistency. In the final stage, findings were reported using descriptive categories, and sub-categories supported by illustrative quotations, as presented in Table 4.

**Table 4:** Inductive coding framework, outlining descriptive categories, sub-categories and illustrative quotations

**Pupil: Identification, Agency and Need**
**1. Early assessment and identification:** “standardised early assessment with a common language”; “gaps in knowing child background”; “current reliance on child to self-identity”;
**2. Children’s voices, agency, and empowerment:** “child having agency”; “what does it feel like for a child?”
**3. Child-centred support, safety, and inclusion:** “how child-centred is the approach/provision”; “is the challenge appropriate for age and stage”; “feeling safe”
**Family: Awareness, Communication, and Collaboration**
**1. Working in partnership with parents/carers:** “schools work with parents”; “transition days for pupils and parents”; “parent/carer workshops”; “support families to understand new culture and practicalities”
**2. Clear communication lines and transparency:** “clear communication at an early stage”; “communicating with families about what to expect, and that there is consistency across secondary schools and transparency”; “parenting newsletter”
**3. Family and generational challenge (social and cultural capital) and adjustment:** “parents’ own experience of school can shape negative experiences”; “being flexible with ‘harder to reach’ families”; “encouraging a positive discourse”
**School: School Partnerships, Cohesion and Continuous Support**
**1. Consistency and transference of knowledge:** “partnerships with parents”; “schools work with continuity”; “start early”; “transition programme defined, not induction”
**2. Familiarity, curriculum continuity, and normalization:** “cross-phase communication”; “use of primary specialists in secondary”; “secondary visible but not overwhelming”; “lack of consistent practice”
**3. National standard for information-sharing and accountability:** “lack of transfer of information”; “transfer of reasonable adjustments”; “national standard for information sharing”
**Local Authority: Cross-Phase Transitions Management, Knowledge Exchange, and Evaluation**
**1. Mapping transitions across Local Authority and facilitating relationships:** “mapping transitions across the Local Authority and a tool to do so”; “agreed collaboration with a guiding framework”
**2. Task group: personnel and chain of command:** “Local Authority transitions steering group”; “Local Authority good practice guide”
**3. Knowledge exchange and personal development:** “menu of support”; ‘is transition trivialized? Local Authority to share research and information and standardise application from Local Authority level”
**National: National Strategy, Accountability, and Communities of Practice**
**1. DfE guidance, national strategy across settings:** “national curriculum for transition”; “statutory guidance”
**2. KPI: what works:** “minimum standard for provision”; “inconsistency of approaches”; “reactive instead of proactive”
**3. National forum for knowledge exchange:** “more guidance for DfE in best practice”; “nation conversation, more awareness and national voice for children”

The descriptive categories developed from the inductive content analysis were then used as a data management tool for analysing the audio data using Thematic Framework Analysis. This is a flexible analytical approach, designed to support researchers to systematically reduce data while making comparisons across codes and delegates (Ozturk et al., 2025; Xu & Zammit, 2020). The following five stages outlined by Ritchie and Spencer (2002) were followed: familiarization (Stage 1), identifying a framework (Stage 2), indexing (Stage 3), charting (Stage 4), and mapping and interpretation (Stage 5).

***Audio data*** The audio recordings of the roundtable discussions were transcribed verbatim by a university-approved external transcriber; then at Stage 1, data were read, and re-read in full by Authors 3, 4, 5, 6 and 7 engaging in a process of familiarisation. Transcripts were then coded using NVivo 14 line-by-line, using the content analysis codes, sub-categories and higher-order conceptual main categories as a data management tool. Any extracts that did not fit optimally into one of these codes were summarised into a new inductive code. Following independent open-coding of one transcript, authors double-coded a second transcript, for credibility. Similarities and differences were discussed, and similar codes were then grouped into categories, which formed the analytical framework, indicative of Stage 2, to organise the data in a meaningful and manageable way. Through a process of indexing (Stage 3) the framework was systematically applied to each transcript to code the data into the framework categories and sub-categories, which were also triangulated for congruence through discussion between all authors to establish confirmability. Charting (Stage 4) was then conducted to organise the coded data into a framework matrix. During the final mapping and interpretation stage (Stage 5), the charted data was synthesised in the context of RQ2 and RQ3 and grounded in examples, providing a coherent, convincing, and authentic interpretation of the data, establishing dependability.

## Results

Key policy messages are presented in turn within each thematic category, alongside supporting example quotations from the audio data transcripts. The thematic framework categories and sub-categories are presented in Table 5.

**Table 5:** A table of the five Thematic Framework Analysis categories and corresponding sub-categories

**Pupil: Identification, Agency, and Need**			
Early assessment and identification	Children’s voices and agency	Child-centred support, safety, and inclusion	Pupil-level anxiety around transitions
**Family: Awareness, Communication, and Collaboration:**			
Develop transitions awareness	Working in partnership with parents/carers	Clear communication between home and school	Family and generational histories and past negative school experiences
**School: School Partnerships, Cohesion, and Continuous Support**			
National standard for information exchange and accountability	School partnerships and communication		Familiarity and continuous support (including curriculum continuity and support needs)
**Local Authority: Cross-Phase Transitions Management, Knowledge Exchange, and Standardisation**			
Mapping transitions across the Local Authority and facilitating relationships	Standardise procedures for sharing information	Sharing and integrating research knowledge into Local Authority policy	A dedicated transitions workforce
**National: National Strategy, Accountability, and Communities of Practice**			
Department for Education transitions guidance and national standards	KPI: what works, that are inclusive in reach and comprehensive in design		National forum for knowledge exchange

N.B. in reference to “National” delegates were referring to facilitators, barriers, and priority areas specifically pertaining to the English educational system, recognising that the aim of the present research is to synthesise views within primary-secondary school transitions educational practice, research, and policy, nationwide, to inform the development of an evidenced-informed primary-secondary school transitions strategy for England

### Pupil: Identification, Agency, and Need

At the pupil level, delegates emphasised the importance of early assessment and identification of individual needs, and recognising children’s voices and agency. Furthermore, acknowledging pupil-level anxiety related to transitions, child-centred support, safety, and inclusion was discussed as integral.

First, ***early assessment and identification*** was considered as essential to ensure that every child within a transferring cohort is supported, and that their unique needs are met. This was reported in terms of the academic side of transitions: *“What I was just mentioning is academic transition and recognising children who finish at ‘Working Towards’ [their expected level] at the end of Key Stage 2. And how you support that, so they don’t end up ‘walking’ [avoiding school] attendance wise”,* and in relation to wellbeing support, with children struggling with the non-academic aspects of transitions, who did not always have a listed support need, often being missed: “*I would say the majority of kids that we work with who could have been helped at transition stage, they’re coming to us at CAMHS [the NHS’s Child & Adolescent Mental Health Services] because they can’t cope with the difference in friendships, different peer relationships. It feels like that’s kind of a stream that tends to get left off”.* Making sure that a range of vulnerabilities are identified as early as possible should be a key priority for a national strategy: *“Whether that be academic, whether that be social and emotional or whether that actually is about…engaging the wider community around that child as well”*, alongside ensuring that supportive interventions are in place from the start of secondary school. One delegate mentioned an example of how this worked at their school: *“On our induction days, we have got an exam assessor basically, and once we’ve highlighted the children, she will be doing a screening test. So, when they come in September [start of school year], their intervention is ready.”*

Secondly, ***children’s voices and agency*** was considered crucially important for successful primary-secondary school transitions, and a mechanism for children’s input, ensuring that they feel listened to and their thoughts and feelings considered, included in any strategy: *“I think pupil voice is probably the most key thing because we often just assume what we think is going to be the best thing to support that young person. We go about organising and planning it all without their input”.* As part of this, delegates emphasised that children need to know what to expect before they reach secondary school, so they can meaningfully contribute to decisions about their support and feel comfortable and engaged: *“Making them feel involved, that they’re contributing to what’s happening, they’re understanding why the support is in place, so that they buy into that”.* This was noted as especially important for children less likely to express their needs, warranting consideration of strategies to encourage communication:* “These young people that aren’t forward, talking about their worries and their anxieties, but are going to need some support. How do you bring them out of their shell, in a way that doesn’t feel like they’re being set apart”.*

Finally, delegates noted that the strategy should also address ***pupil-level anxiety related to transitions****,* emphasising the importance of ***child-centred support*** that prioritises ***safety and inclusion*.** Central to this in their view was practitioners' active engagement with children to explore their personal understanding of safety, belonging, and inclusion: *“Another thing that I think is safe – what does it take to be safe? That’s a big one, you know, they want to feel safe, don’t they? And children go to school, and they don’t feel safe, so actually, it’s unpicking what that means.”* Reframing concerns and promoting a more balanced discourse surrounding primary–secondary transitions was discussed as a key priority: *“They’re going to have a fear element because it’s new relationships, new attachments, new friends and stuff. But through wellbeing curriculum, you can draw on that strength and resilience aspect to really change the viewpoint for the child.”* Equally, normalising anxiety was considered essential, recognising it as an expected component of transitions and emphasising the importance of sustained support: *“Building resilient children is partly owning the fact that life is not always stress-free […] no one’s said to them that being worried and anxious about this is okay. We’ll walk you through it, but you are going to be anxious about it.”*

### Family: Awareness, Communication, and Collaboration

At the family level, developing awareness, clear and accessible communication methods between home and school, and working in partnership with parents/carers, acknowledging family and generational histories and past negative school experiences, was discussed as important.

Families were frequently noted as a vital part of the transitions process, and engaging them and the child together, as part of the same journey, to ***develop transitions awareness*** integral: *“I think that’s what it’s got to be about—the parents and the child joining the new school, transitioning together”.* This was seen to be especially important when parents/carers have strong emotions towards the process that their child may not share: *“I hear a lot that the kids are fine, the parents are anxious”* which may inadvertently be passed onto the child, leading, in the extreme, to school avoidance: *“They don’t know which way to turn, that anxiety is then passed onto the young people […] what we’ve found this summer is loads of Year 7 s not taking their places”.* Subsequently, ***working in partnership with parents/carers***, across the whole transitions process, not only when something negative happens, was considered the most effective way to sustain engagement and ensure that schools and families work together proactively for the best outcome for the child: *“We need to start in that preventative space rather than a reactive space. As soon as it’s reactive, parents won’t engage. They’re more likely to think, ‘oh, you’re doing this to help me and help us, we’re going to move forward’ as opposed to ‘it’s because my child has done something wrong, so you’re just basically telling me off”.*

***Clear and accessible communication between home and school*** was also reported to be important: *“As a family, knowing who you can talk to, having a very clear person, a first point of contact that you can rely on*”*.* Getting information to those families who need it the most was reported as challenging: *“The parents who really want to find out the information are the parents who’ll turn up. It’s how we get to parents who possibly need it more”.* Indeed, *“Schools sometimes label their parents, ‘oh, she doesn’t care. He doesn’t bother’ And I actually think those are the ones we need to work harder at”* and identifying these families was proposed to be a key element of a transitions strategy. As part of this, they highlighted the importance of acknowledging ***family and generational histories and past negative school experiences*** as these can influence parental attitudes and the messages conveyed to children, creating barriers to smooth transitions: *“Parents who themselves, as children, had negative experiences of secondary school—that then shapes their whole discourse around what secondary school is, shapes their communication with their child about it and that can possibly set the child off on a particular path”.*

### School: School Partnerships, Cohesion, and Continuous Support

The delegates noted that integral to “gold-standard” transitions provision at the school level is school partnerships and communication, consistency in the transference of knowledge and information, curriculum continuity and familiarity.

Delegates reported that one of the largest barriers of “gold-standard” transitions at the school level is the *“transfer of information, so communication between primary and secondary”.* Therefore, they noted that attention should be given to developing an appropriate ***national standard for information exchange and accountability***. This could mirror the policy Wales have adopted: *“Since 2022, all secondary schools have to have a transition plan working with all their cluster primary schools and they have to then share that and work with their cluster primary schools to support the transition”.*

They also noted that individual and broader strategies should be a part of this plan, and at the child level, there should be a balance between ensuring that secondary school teachers know about a child’s potential behavioural, emotional or wellbeing challenges so that ***continuous support*** can be provided, and making sure that they are afforded an opportunity for a ‘fresh start’, where they are not followed into secondary school by a negative label: *“If a child, in primary school, has had a lot of behavioural difficulties, do we say to the child, ‘don’t worry. Clean slate, start afresh—nobody knows you and you can start all over again’. The child might find that reassuring, however, what’s happened to all the information about how primary teachers have managed the behaviour and the strategies they’ve put in place? That’s absolutely valuable information”.*

This complexity can be compounded when a secondary school has multiple feeder primaries, all with different ways of recording and transferring information about their pupils, and different working relationships with the secondary school. Thus, consistency and ***school partnerships and communication*** was considered vital within such collaboratives: *“The discrepancy between that transfer of information when you’ve got like five different primary schools going into one secondary school […] and it’s that inconsistency that doesn’t help because some children will have a really good transfer and some others won’t, and they might be going into the same school; it depends on school relationships”.*

Schools providing ***curriculum continuity*** was also discussed as important: “*We are trying to create some sort of middle ground, so that transition between primary curriculum and secondary curriculum is kind of gelled together. We have taken a bit of time to have a look at the secondary curriculum and there is quite a leap”.* At a cohort-wide level, transition days were commonly mentioned, as helpful to develop ***familiarity,*** however a barrier to transitions outreach was capacity: *“Just for the staff from the high schools to get out and do all those visits; capacity has been a real issue”.* A national priority for these outreach events, including transitions days, could address these capacity issues. They argued that this could ensure that events are properly funded, staffed, and sufficiently prepared for in advance across feeder networks, LAs, and nationally.

Finally, delegates noted that aligning strategies and interventions with their existing goals and standards, is vital if schools are to adopt Local Authority and/or Trust transitions policies or strategies: *“A local strategy, which is developed with schools and you’ve got the buy-in from schools, is always going to be more effective than something that people just see as a bit of an add-on being imposed upon them”.*

### Local Authority: Cross-phase Transitions Management, Knowledge Exchange and Standardisation

Mapping transitions across the Local Authority and facilitating relationships; research, knowledge exchange and evaluation; and a dedicated transitions workforce, was discussed as integral for “gold-standard” transitions provision at the Local Authority level.

Cross-phase transitions management at the Local Authority level, by ***mapping transitions provision across the Local Authority and facilitating relationships***, was seen to be integral to ensuring that all schools are working together with a shared strategy, towards a shared outcome: *“So basically, you’ve got a mix of primary schools – lots of small primary schools, village primary schools who are not academies yet. But they’re all working together in little clusters. So, the academies plus the primary schools are working together and that’s collaborative. So, I think the brokerage is getting there”.*

Part of this collaboration, according to the delegates, should include ***standardised procedures for sharing information*** about pupils across transitions, and the general importance of ***standardised procedures*** was noted within the Local Authority: *“You need those standardised processes across a geographical area, and yeah, the forms go with it and how you share information, but it’s having that agreement and expectation, that’s just the main thing”.* Having a mandatory system for exchanging pupil information, with a ***standardised criteria*** was discussed as important: *“the information all has to be shared of all the children *etc*. […] And then there’s a requirement in the policy that they then have a visit to each of the primary schools to discuss the children”,* agreed and managed by the Local Authority to provide accountability: *“It’s in agreement across the whole Local Authority area now that every school has to do that, academy or not […] it will assist with so many problems that it’s worth it for them to do that administration”.*

***Sharing and integrating research knowledge into Local Authority policy*** was discussed as vital. Recognising that Local Authorities could provide the most logical forum and organisational support: *“I think what LAs need to do is put the research on the table: now more than ever. Scream it out loud to get people to understand because I think transition is trivialised, I’m afraid […] the research says everything and I think Local Authorities need to use it more […] and say, ‘This needs to happen’”.* However, this would only work if done carefully and delivered by a trusted figure: *“I think that’s really important as well, but unless it comes strategically, it isn’t going to get there unless a particular headteacher or an executive head or a cluster or an academy trust really believe in it”.* This credibility, in their view, could be achieved by integrating knowledge at a national level, with a focus on measurement of impact to demonstrate success: *“So, you spent £1500 on this? And what’s the impact on the young people? Has it made a difference to them, their parents…?’”.*

A ***dedicated transitions workforce,*** who have a range of expertise across transitions was discussed as important: *“I think it’s the Local Authority getting back to having the right staff in their transition teams”.* Core to this idea is the unification of transitions as a tangible concept that requires distinct processes, policy, and attention, rather than being scattered across other elements of school policy, although challenging: *“One of the challenges I find in the Local Authority is actually, lots of teams do things on transition, but actually, drawing that together as a local strategy is key, although a challenge for somebody to take on”.* A national strategy that includes this could provide the resources needed to enact this at a Local Authority level.

### National: National Strategy, Accountability, and Communities of Practice

To realise a national approach, delegates discussed the importance of Department for Education (DfE) guidance and national standards, which are inclusive in reach and comprehensive in design, with key performance indicators and a national forum for knowledge exchange.

Delegates reported the importance of having ***Department for Education transitions guidance***, which would provide credibility to the work that schools are doing, set consistent ***national standards*** and showcase approaches that have worked across the UK: *“What we’re trying to develop is like a quality mark, as it’s something that schools desire and want. I would love it if the DfE provide guidance […] We want to be able to give schools ideas – these are the things that are happening round the country and they’re really, really good”.* The lack of conversation surrounding transitions in national government conversation was also noted as a concern:* “I think just having a national conversation about transition in general because, you know, there’s nothing about transition in the Children’s Wellbeing Bill […] when you look at that bigger picture, there’s the conversations around SEND, there’s the conversations about school absence, but where does transition feature?”.*

They highlighted that any approach designed should be **inclusive in reach and comprehensive in design**: *“At Westminster level, they need to value transition, and they need to adopt a holistic approach, an ecological perspective”.* To ensure a national strategy is applicable across as many Local Authorities and schools, they noted that it should primarily address the most critical and widely shared issues related to transitions, which need to be identified: *“Our key priority is finding out what the key priorities are! […] I’ve got four or five at the moment. But is one more important than the other? I don’t know”.*

Furthermore, a strategy should include ***Key Performance Indicators (KPIs)*** underpinned by what works: *“To get buy-in from Head Teachers, you need to show what the advantage is going to be for them”*, and include focus on wellbeing, in addition to more quantifiable outcomes like attainment and attendance: *“Those absence rates, those mental health rates for children and young people. Attendance is going down, and their mental health is going down, you know, those are kind of national concerns, but you need a national expectation, don’t you? Yeah, it sets the expectation—your LA implement it”.*

A **national forum for knowledge exchange**, where “gold-standard” provision could be disseminated from a national to a Local Authority to a school level was discussed as essential: *“If you have that sort of national goal, how do we make sure that best practice, things [that are mentioned] round these tables, get out to schools?”,* e.g. through a regular national conference as a means of disseminating best practice: *“Maybe an annual conference for more best practice sharing, such as this one”.*

## Meta-inferences and Recommendations

To maximize the scientific rigor, efficiency, and yield of mixed-methods research, it is integral that mixing of methods is purposeful in data generation, analysis, and interpretation. However, many mixed-methods studies fall short in describing the analytical framing and procedures used, in addition to the process of generating and presenting meta-inferences. A meta-inference, according to Tashakkori and Teddlie (2008, p. 101), is an “overall conclusion, explanation, or understanding developed through an integration of the inferences obtained from the strands of a mixed methods study”. How the strands are merged within this depends on the rationale of the research, and for the present study, a simultaneous bidirectional approach was taken (Moseholm & Fetters, 2017). This means that the findings from each element of the research were regarded as equally important for our aims, and although analysed separately at first, they were combined in a “back and forth” manner, revisiting them iteratively as findings emerged.

Furthermore, to date, research on educational transitions is often not being accessed or used by educators (Edge et al., 2024), with practitioners typically relying on anecdotal evidence or informal networks to shape practice (Bagnall et al., 2024a). This has contributed to ‘patchwork provision’ across schools, Local Authorities, and regions, leading to significant inconsistencies in the quality, scope, and impact of transitions support (Bagnall et al., 2020, 2024a; White, 2020). Given the time and financial pressures facing schools, and the risks associated with poorly managed primary-secondary school transitions, there is a pressing need to disseminate concise, evidence-informed, and practically applicable guidance to support educational practitioners in strengthening transitions provision.

To overcome this gap, the present study utilises a pragmatic approach, valuing shared meaning and joint action. Following an exploratory sequential mixed-methods design, Study 1 informed the conceptual focus and development of Study 2, while maintaining analytic independence between the two strands prior to integration. As outlined above, each data strand was first analysed separately in relation to the specific RQs it addressed, ensuring the internal coherence and methodological rigour of each study and supporting the development of a comprehensive understanding (Bagnall et al., 2024a; Demkowicz et al., 2023).

**Fig. 2:**
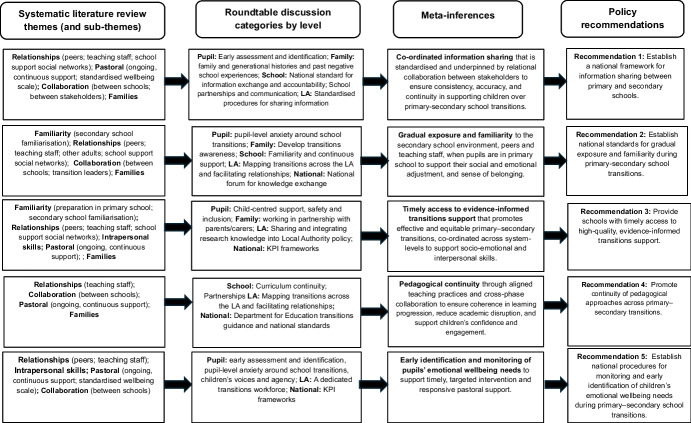
Visual merging matrix presenting findings from the systematic literature review, roundtable discussions, meta-inferences and policy recommendations

Following this independent analysis, findings were integrated by Authors 1, 2, 4, 5, 6, 14 and 17 using a simultaneous bidirectional approach (Moseholm & Fetters, 2017), with cross-checking for quality assurance and to reduce interpretive bias. The materials for integration comprised the final thematic outputs from each strand: six themes and 12 sub-themes from the systematic literature review, and five thematic framework categories and 18 sub-categories from the roundtable consultation. This stage brought the two strands together to support complementarity, enhance interpretive depth, and generate meta-inferences that extend beyond the contribution of either study alone (Feilzer, 2009; Morgan, 2014). To do this, a visual merging matrix was created, juxtaposing the findings from the data sources to draw meta-inferences, which is presented in Fig. 2 for transparency, with each inference linking to its underpinning evidence from both studies to ensure traceability. This process enabled identification of convergence, complementarity, and divergence. Predefined decision rules were applied: convergent findings were treated as higher-confidence evidence; complementary findings were integrated to extend interpretation; and divergent findings were retained and explored to identify contextual explanations, which were iteratively discussed drawing on the original data extracts.

Meta-inferences were then synthesised by Author 1 and Author 17 and cross-checked by the full project team for quality assurance, as a list of actionable recommendations with key actions outlining illustrative examples of what “gold-standard provision” could look like in practice. The full project team brings substantial collective expertise in research, policy, and practice across all four nations of the UK, and this breadth of expertise and policy-contextual knowledge ensured that the recommendations were carefully grounded in the realities of policy and practice in England, supporting the development of outputs that are not only evidence-informed but contextually situated and actionable.

It is important to note, however, that whilst the recommendations are grounded in and traceable to the integrated evidence, the move from meta-inferences to specific policy recommendations involved, by necessity, an interpretive step. As such specific implementation proposals, such as references to statutory mechanisms, KPI structures, digital platforms, and accountability arrangements, represent illustrative examples of how recommendations might be enacted in practice, rather than direct empirical conclusions drawn from the data alone. This is an inherent and acknowledged feature of applied, stakeholder consultation, and is made explicit here in the interests of transparency. These recommendations are presented in detail in Table 6, and listed below, with examples of what some key actions may include:

**Table 6:** Policy Recommendations for a primary-secondary school transitions strategy in England

Policy Recommendation 1
Establish a ***national standardised framework for information sharing*** between primary and secondary schools to ensure consistency, accuracy, and continuity in supporting children over primary-secondary school transitions. This framework should outline the essential data to be shared, timelines for transfer, and protocols for collaboration between schools ***Key actions may include:*** • Developing a **national information-sharing template** outlining key academic, wellbeing, friendship and socio-demographic data to accompany each child. It is important that the child and families have an input in this, and consent to data sharing • Creating a **centralised digital platform** to securely facilitate and standardise data exchange between schools and across Local Authorities • Encouraging **joint transitions meetings** between primary and secondary staff to discuss individual needs, particularly for vulnerable learners • Introducing **mandatory timelines** for data transfer to ensure secondary schools receive relevant information in advance of transitions planning • Embedding **accountability measures** to monitor compliance and the quality of information sharing at both school and Local Authority levels
Policy Recommendation 2
Develop ***national standards for facilitating gradual exposure and familiarity during primary-secondary school transitions*** to support children’s emotional and social adjustment over time. These standards should aim to reduce logistical and administrative barriers that limit opportunities for primary school children to engage with their future secondary schools ***Key actions may include:*** • Introducing earlier and more frequent transitions visits (e.g., beginning in Year 5 or equivalent to use the gym at secondary school etc.) • Designated days at the start of term where only Year 7 and a buddying year (e.g. Year 9) attends, to support integration and confidence-building • Establish **nationally co-ordinated transitions visit days** across Local Authorities to ensure greater consistency of practice and equity in transitions experiences for children • Providing an **online transitions package** for parents/carers and children (including virtual tours, staff introductions, and peer relationship guidance when children are in the last year of primary school) • Encouraging **innovative, locally adapted approaches** to enhance children’s familiarity and sense of belonging prior to their transfer to the -secondary school
Policy Recommendation 3
Ensure that all schools have ***timely access to high-quality, evidence-informed transitions support that*** promotes effective and equitable primary–secondary transitions. This provision should align with **national pastoral priorities**, include **mechanisms for sharing best practice** across schools and Local Authorities, and embed transitions-related criteria within **Ofsted inspection frameworks** to strengthen accountability and consistency ***Key actions may include:*** • Designing a **national primary-secondary school transitions skills-based curriculum** which develops children’s awareness, knowledge and ability to manage the multiple changes they will experience over primary-secondary school transitions, through scaffolding socio-emotional and interpersonal skills across, at least, the last two years of primary school and first year of secondary school • Developing a **national repository of evidence-informed primary-secondary school transitions resources and case studies**, accessible to all schools and Local Authorities • Establishing **regional communities of practice networks and partnerships** to facilitate knowledge exchange, sharing and scaling of effective primary-secondary school transitions practices • Providing **professional development opportunities** for school staff on implementing and adapting primary-secondary school transitions curricula to meet children’s needs on a universal and targeted basis. This could also be included in Initial Teacher Education programmes • Embedding **family engagement and support** as an integral component of transitions planning to keep parents and carers informed, prepared, and involved • **Formalise minimum practice standards** for primary–secondary transitions for both primary and secondary schools through legislation or statutory guidance, e.g. **embed transitions planning and support** within school improvement and pastoral care frameworks, **promote transitions focused quality marks**, and/or **include transitions quality indicators** in Ofsted criteria focused on wellbeing, inclusion, and transitions preparedness
Policy Recommendation 4:
Promote **continuity of pedagogical approaches across primary–secondary transitions** to ensure coherence in learning progression, reduce academic disruption, and support children’s confidence and engagement. A nationally guided but locally adaptable framework should align curriculum expectations, pedagogical approaches, and assessment practices across Key Stages 2 and 3 ***Key actions may include:*** • Developing **national guidance on curriculum alignment** between primary and secondary phases, particularly in core subjects such as English, mathematics, and science, and **include curriculum continuity indicators** within evaluation and accountability frameworks (e.g., Ofsted) • **Promote cross-phase curriculum collaboration** by encouraging joint planning and moderation between Year 6 and Year 7 teachers, and **cluster-based partnerships** to develop shared teaching resources, coherent progression frameworks, and cross-phase projects • Providing **professional development opportunities** focused on transitions pedagogy and curriculum bridging strategies • Embedding **formative assessment practices** that recognise prior learning and identify gaps early in the secondary phase • Encouraging **family and child knowledge exchange** to foster a shared sense of academic progression and purpose
Policy Recommendation 5:
Develop a **national approach to monitoring children’s emotional wellbeing and ensuring early identification of emotional needs** to promote timely, targeted support during primary–secondary school transitions. A consistent framework would enable schools to identify emerging concerns, intervene proactively, and reduce the escalation of mental health difficulties ***Key actions may include:*** • Establishing a **nationally standardised wellbeing monitoring framework** for use across all primary and secondary schools, incorporating validated assessment tools, with embedded **routine emotional wellbeing screening** at key transitions periods (e.g., across Year 5, 6, 7 and 8) • Integrating emotional wellbeing indicators into **school self-evaluation and Ofsted inspection criteria** to promote accountability • **Build staff capacity and early intervention systems** by training school staff to recognise and respond to early signs of poor emotional wellbeing**,** ensuring **clear referral pathways** and **timely access** to early help services for children identified as at risk • Create structured and proactive processes across primary and secondary schools that involve **family engagement, partnership and communication** to support children’s emotional wellbeing during primary-secondary school transitions

**Recommendation 1:** Establish a national framework for information sharing between primary and secondary schools:

- Specifying the essential data to be shared;
- Developing an information-sharing template;
- Introducing mandatory timelines and protocols for schools;
- Creating a centralised digital platform to securely facilitate and standardise data exchange between schools and across Local Authorities.

**Recommendation 2:** Establish national standards for gradual exposure and familiarity during primary-secondary school transitions:

- Introducing nationally co-ordinated transitions visit days across Local Authorities;
- Holding earlier and more frequent transitions visits (e.g., beginning in Year 5);
- Designating specific days at the start of the academic year where only Year 7 pupils and a buddying year (e.g. Year 9) attend;
- Mandating levels of minimum practice for schools to enhance parents/carers and children’s familiarity with their next chapter.

**Recommendation 3:** Provide schools with timely access to high-quality, evidence-informed transitions support:

- Designing a national primary-secondary school transitions skills-based curriculum;
- Creating an open-access national repository of evidence-informed primary-secondary school transitions resources and case studies;
- Establishing regional communities of practice networks and partnerships to facilitate knowledge exchange;
- Providing professional development opportunities for school staff;
- Embed family engagement and support in transitions planning;
- Formalise minimum practice standards for primary–secondary transitions planning and support.

**Recommendation 4:** Promote continuity of pedagogical approaches across primary–secondary transitions.

- Developing national guidance on curriculum alignment between primary and secondary phases;
- Encouraging cross-phase curriculum collaboration between Year 6 and Year 7 teachers;
- Providing professional development opportunities for school staff;
- Embed formative assessments in Year 7 to track prior learning and identify gaps;
- Encourage family–child knowledge exchange to support shared progression.

**Recommendation 5:** Establish national procedures for monitoring and early identification of children’s emotional wellbeing needs during primary–secondary school transitions:

- Establish a nationally standardised wellbeing monitoring framework for use across schools at key transitions points;
- Integrating emotional wellbeing indicators into school self-evaluation and inspection criteria;
- Build staff capacity for early identification and intervention, with clear referral pathways to early help services, and involve family engagement;
- Commissioning research to identify emerging concerns, intervene proactively, and reduce the escalation of mental health difficulties.

## Discussion and Conclusion

In England there is pressing empirical and practical need to co-develop an evidence-informed, system-wide strategy for improving primary-secondary school transitions. To do this, the present research innovatively triangulates findings from academic research, with extensive stakeholder consultation to develop an evidence-based framework with key recommendations. This offers a conceptual foundation that may help inform the development of an evidenced-informed primary-secondary school transitions strategy in England, while also contributing to the development, enactment, and evaluation of robust primary-secondary school transitions research, and high-quality practice, addressing some of the existing limitations within the field. In this section, practical and contextual challenges associated with introducing a primary-secondary school transitions strategy in England, will be considered, such as resource disparities, data governance, and maintaining local flexibility. In addition, directions for future research, including how policy design and introduction coherence can strengthen equity and consistency in children’s experiences of primary-secondary school transitions, will be discussed.

Primarily, the present study highlights the need for coordinated, system-wide action over primary-secondary school transitions that recognises interrelated influences at the pupil, family, school, local authority, and national levels. This is embedded across the five policy recommendations, where emphasis is placed on developing relationships, sustained inter-school collaboration and partnerships, communities of practice, and active engagement with families, to facilitate knowledge exchange, information sharing and scaling of effective primary-secondary school transitions practices. The significance of collaborative support over primary-secondary school transitions between school systems and stakeholders, has also been raised consistently in previous research (Bagnall et al., 2020; Jindal-Snape et al., 2021). However, known academic, social, and environmental discontinuities across primary and secondary school systems can represent barriers, leading to fragmented support, poor information sharing, disjointed continuity of pedagogical approaches and curriculum continuity, and declines in feelings of school connection and wellbeing (Mumford & Birchwood, 2021), which was similarly shown in the present research.

In part, challenges in coordinated, system-wide action over primary-secondary school transitions may stem from inadequate conceptualisation of transitions as a one-off “event”, as opposed to an ongoing process of adaptation across domains and contexts (Jindal-Snape, 2016, 2023), which was shown in both the systematic literature review and round-table discussions. This narrow framing has contributed to the development of policies and practices that are not fit for purpose (Hannah et al., 2023; Jindal-Snape et al., 2020; Symonds et al., 2023). For instance, considering primary-secondary school transitions in line with the former, support could involve a single visit or handover meeting, rather than as a continuous, planned process encompassing curriculum alignment, pastoral support, and structured parent/carer communication and engagement (Jindal-Snape, 2023). The same applies in relation to the design and operationalisation of instruments to assess children’s emotional wellbeing in the context of primary-secondary school transitions (Recommendation 5), which are often not sensitive and stable enough to capture the complex changes children negotiate during primary-secondary school transitions (Bagnall et al., 2024b). As a result, many instruments are operationalised at one time point pre and post the “move”, as opposed to taking a longitudinal approach, e.g., over the last two years of primary school and first two years of secondary school (Bagnall & Jindal-Snape, 2023; Jindal-Snape & Cantali, 2019). Such short-term approaches risk generating misleading data, and in turn, suboptimal transitions policies and practices (Bagnall & Jindal-Snape, 2023). Therefore, it is essential that policy makers and practitioners critically engage with research literature and work collaboratively with researchers through co-production to ensure that transitions policies and practices are both evidence-informed and grounded in real-world practice. This underscores the importance of ongoing professional development and research capacity building to strengthen the reciprocal flow of knowledge between research, policy, and practice. It also highlights the responsibility of researchers to disseminate their research in ways that are accessible to different stakeholders. For instance, a comic anthology was created to share the research findings of a primary-secondary school transitions study in an engaging and accessible manner not only for professionals but also for children and their families. It includes tips from pupils for other pupils and provides guidance on using the anthology as a resource in the class and home (Jindal-Snape et al., 2023).

These conceptual limitations not only demonstrate the abject nature of the current field with respect to key assumptions (i.e., transitions as a static timepoint) but also have significant consequences for identifying and supporting children’s emotional wellbeing during this time, which has been acknowledged as a pressing concern (Moore et al., 2021). To realise a primary-secondary school transitions strategy in England, work is needed to first identify a robust and feasible measurement strategy to understand (a) which aspects of primary-secondary school transitions children are experiencing emotional difficulties with; (b) who might be particularly vulnerable; and (c) what universal and targeted support could be useful (Bagnall et al., 2024b). This is recognising that while school inspections (e.g., Ofsted in England) may assess transitions as part of broader pastoral and curriculum reviews; a significant challenge within primary-secondary school transitions research and practice is that there is no national mechanism in place to effectively monitor children’s emotional wellbeing during primary-secondary school transitions, regularly and consistently. This is despite urgent calls within primary-secondary school transitions literature to systematically monitor children’s emotional wellbeing in the context of primary-secondary school transitions longitudinally, recognising the steady declines shown during this time (Bagnall et al., 2025a; Beatson et al., 2023; Donaldson et al., 2024). This is particularly timely in the context of an amendment to the Children’s Wellbeing and Schools Bill progressing through UK parliament, which proposes national monitoring of children’s emotional and mental health (UK Parliament, 2025).

However, as shown in Bagnall and Jindal-Snape’s (2023) systematic literature review, which synthesised primary-secondary school transitions papers published between 01/2008 and 03/2021 that had used child self-report measures to assess school transitions experiences and/or emotional wellbeing and/or factors believed to underpin emotional wellbeing, existing measures demonstrate several limitations. These include: (a) inconsistent reliability and validity (Akos, 2002); (b) align with a predominantly negative discourse using unbalanced items and negative terminology (Thomasson et al., 2006); (c) lack clear conceptual and theoretical grounding (Jindal-Snape et al., 2021); (d) lack a longitudinal focus in their design and operationalisation (Rice et al., 2011); and (e) critically, there is no existing scale which comprehensively assesses children’s emotional wellbeing in the context of primary-secondary school transitions (Bagnall et al., 2025b). The lack of a robust scale that measures children’s emotional wellbeing in the context of primary-secondary school transitions is limiting the field’s ability to generate robust, comparable, and contextually meaningful evidence to inform research, practice, and policy, as shown in the inconsistencies in measurement approaches, difficulties in benchmarking outcomes across studies, and challenges in identifying and evaluating effective transitions support (Beatson et al., 2023; Bharara, 2020).

Together this body of research calls for the need for future research to develop a single, up-to-date, robust and reliable scale. Overcoming previous limitations within the field, standardised monitoring tools need to be contextually relevant to children’s lived experiences, environments, and conceptualisations of emotional wellbeing in the specific context of primary-secondary school transitions (Bagnall & Jindal-Snape, 2025), as raised by researchers (Beatson et al., 2023), practitioners (Bagnall et al., 2025a), and children (Bagnall et al., 2024a, b; Demkowicz et al., 2023). To do this standardised monitoring tools need to be developed in partnership with primary-secondary school aged children to ensure that items are meaningful and holistically measure contextual experiences during this time, and the language and format is also age and stage appropriate, following a sensitive balanced discourse. One potential instrument that could address this gap, is the *Primary-Secondary School Transitions Emotional Wellbeing Scale* (*#P-S WELLS*) which has recently been co-developed with children (aged 9–13 years), educational and clinical practitioners*,* NGOs and policy makers to assess children’s emotional wellbeing in the context of the social, academic, environmental and personal changes they are negotiating over primary-secondary school transitions (Bagnall et al., 2024b). Policy makers may wish to consider engaging in the validation, implementation and rollout of contextually sensitive instruments, with *#P-S WELLS* being an illustrative example. A coherent measurement framework could potentially contribute to the data infrastructure needed to ensure early identification of children’s emotional needs in the context of primary-secondary school transitions and may help to inform the development of evidence-based norms. This recognises that at present, there are no consistent England-based performance indicators or datasets which have specifically monitored children’s emotional wellbeing over primary-secondary school transitions. In turn, this could inform policy decisions and resource allocation to drive sustained improvements in primary-secondary school transitions provision, through identifying and refining best practices, and guiding early intervention efforts.

However, while early identification is crucial, it must be matched with sufficient access to appropriate resources, interventions and services; otherwise, schools may face increased demand without the means to respond effectively, which Policy Recommendation 3 directly addresses. To ensure effective and equitable primary–secondary transitions provision, educators need timely access to high-quality, evidence-informed transitions support provision, which has not only been acknowledged in the present research, but also by children (Bagnall et al., 2024a); their parents/guardians (Bagnall et al., 2020); teachers (Jindal-Snape et al., 2021); and within education policy (Department for Education, 2023). This could be achieved by a national body whose remit is to co-ordinate research, identify best practice and to disseminate information directly to practitioners. Such a body could draw upon the knowledge from all four nations, sourced from a combination of academics, practitioners and parents/carers.

Furthermore, given the time and resource pressures schools face, a primary-secondary school transitions curriculum (as outlined in Recommendation 3) could address this gap, by providing a consistent framework, incorporating measurable objectives, quality markers, and structured support, to help reduce disparities in implementation across schools and regions, by setting minimum standards (Evangelou et al., 2008; Bagnall & Stevens, 2026). This has been advocated by educators in previous research: “I would really like to see some sort of curriculum expectations in Key Stage 2, focused on developing skills, and having a skills-based transition curriculum” (Bagnall et al., 2024a, p. 10).

Finally, it is essential that a standardised framework for information sharing between primary and secondary schools is established to ensure consistency, accuracy, and continuity in pupil support, as outlined in Recommendation 1. A standardised information-sharing framework should clearly define the core data to be shared, the timelines for transfer, and the protocols for collaboration between schools, ensuring that information exchange is systematic, equitable, and securely managed. However, it is important to acknowledge potential limitations, including variability in school resources, digital infrastructure, and data management systems, as well as concerns regarding data protection and workload implications for staff. Therefore, this framework should be co-produced with educators to balance practicality, ethical considerations, and meaningful impact, ensuring that the resulting system is both evidence-informed and feasible within the realities of school practice.

## Strengths and Limitations

The present research has several strengths. First, this paper demonstrates methodological rigour by employing a transparent and replicable analytic process that integrates systematic literature review insights with stakeholder perspectives. Through this empirical precision and depth, this paper advances understanding of how best to support primary-secondary school transitions within practice, providing a robust conceptual foundation to inform the development, introduction and evaluation of policy and practice. The inclusion of 52 national experts from education practice, policy, and research strengthens the credibility, relevance, and reach of the findings, ensuring that recommendations are both evidence-informed and have practical utility, ultimately demonstrating the viability of meaningful co-production.

However, there are also limitations. First, although the systematic literature review provided a robust foundation for identifying key priority areas, systematic literature reviews in general share several common limitations, pertaining to the quality and scope of available studies, which may have introduced publication or selection bias, never being fully up-to-date, given the time lag between the research being conducted and the paper being published. Second, the round-table discussions, while rich in expert insight, relied on a purposive sample of 52 experts within educational practice, research, and policy, nationwide. While many experts were also parents, obtaining the perspectives of parents and particularly children themselves, especially from unrepresented groups, would have enhanced the usefulness of this strand of data collection. Finally, while the integration of literature and expert perspectives provides strong meta-inferences, these findings remain primarily conceptual; further empirical testing is required to evaluate the feasibility and impact of the proposed recommendations in practice. Such future work should adopt a longitudinal, mixed-methods and co-production approach, engaging educators, policy makers, children, and families to ensure that the resulting transitions practices are not only evidence-informed but also contextually relevant and feasible to roll-out. This will be critical to establishing a robust, enduring framework with both national coherence and local flexibility, ensuring that the current recommendations translate into measurable, long-term impact, fostering more consistent, equitable, and effective transitions provision across England, and providing a template for countries seeking to do the same internationally.

## Concluding Statement

In sum, this is the first study to synthesise methodological approaches, triangulate multiple stakeholder perspectives and multidisciplinary evidence to develop an evidence-based framework with actionable policy recommendations to improve primary-secondary school transitions in England. This offers a conceptual foundation for developing an evidence-informed primary–secondary school transitions strategy for England, while also providing a robust framework to guide the design, enactment and evaluation of high-quality transitions research and practice internationally, thereby addressing long-standing limitations within the field and supporting more coherent, equitable and impactful approaches to transitions. This study has a lot to offer other countries who might be interested in developing a national primary-secondary school transitions strategy, demonstrating international, as well as national reach.
